# Headroom based adaptive droop control for regulating DC voltage and active power in MTDC grid with integrated renewable energy

**DOI:** 10.1038/s41598-026-38678-2

**Published:** 2026-02-07

**Authors:** Zi-Hong Jiang, Asif Raza, Yi-Die Ye, Muhammad Punhal Sahto, Ghalib Raza, Malik Haris, Muhammad Shahid Mastoi, Mannan Hassan

**Affiliations:** 1 School of Information Science and Engineering, NingboTech University, Ningbo, 315100 China; 2 College of Information Science and Electronic Engineering, Zhejiang University, Hangzhou, 310027 China; 3 Department of Mechanical Engineering, NFC Institute of Engineering and Technology (IET), Multan, 60000 Punjab Pakistan; 4 Department of Electronics Engineering Technology, The Benazir Bhutto Shaheed University of Engineering Technology and Skill Development (BBS-UTECH), Khairpur Mirs, 66020 Sindh Pakistan; 5 Interdisciplinary Research Center for Sustainable Energy Systems, King Fahd University of Petroleum & Minerals, Dhahran, 31261 Saudi Arabia

**Keywords:** Energy science and technology, Engineering

## Abstract

Active power and DC voltage regulation is the key control challenge in a voltage source converter based multi-terminal high voltage direct current system (VSC-MTDC), particularly when integrating renewable energy sources. In such a system, the droop control may result in VSC overloading, leading to unequal power distribution and DC voltage instability following a major disturbance. To enhance the control performance, this paper presents an adaptive droop control technique based on the headroom of VSC (HR-ADC). The designed approach uses the headroom of rectifying and inverting converters to adaptively adjust values of droop coefficient, thereby avoiding converter overloading when significant power interruptions occur. The planned strategy is adopted for the rectifying and inverting converters. To validate its effectiveness, the HR-ADC is compared with the variable droop control using the four terminal ± 400 kV MTDC transmission system in PSCAD. Simulation results demonstrate that the designed HR-ADC is autonomous and resilient, ensuring stable system operation even during significant disturbances such as converter outage or fault conditions in the MTDC grids.

## Introduction

### Background

High voltage direct current (HVDC) technology has been extensively used to integrate the renewable energy (RE) based power generation and to facilitate the effective exchange of power with conventional power grids to enhance power transmission capacity and reduce power losses^1^. Typically, HVDC systems are implemented in monopolar and bipolar configurations which restrict their scalability and limit broader deployment in complex power networks^2^. The shortcomings of monopolar and bipolar HVDC systems have been revealed by the recent development with regards to operational flexibility and scalability. Multi terminal HVDC (MTDC) systems using voltage source converters (VSCs) design have been developed to address these issues^3^. This grid allows connecting multiple conventional AC (alternating current) grids, RE based power generation, and provides a better flexibility of operation, dynamic power exchange, and modified fault tolerance^4^. Its main advantages include cost effectiveness and flexible power control, making it ideal for large scale RE integration for modern power systems^5^. As a result, it has been identified that MTDC networks can improve grid reliability and accommodate the dynamic nature of renewable generation^6^. However, the growing complexity of MTDC networks requires robust control schemes to ensure stable and reliable operation. The instability of the system caused by the rapid imbalanced power allocation among VSCs in response of major disturbance can lead to substantial DC voltage fluctuations that may risk the stability of the system^7^. Hence, DC voltage regulation with stable limits is essential to maintain the secure and reliable operation of the entire MTDC grid^8^.

### Literature review

In response to DC voltage and active power instability challenges in MTDC grid, several control techniques have been proposed to improve stable and consistent performance of the system. Hierarchical control^9^ and model predictive control^10^ are advanced methods used to enhance control effectiveness. However, the design of these techniques are typically computed depending on the rated capacity of the converter. During a significant power disturbance, they may not prevent DC voltage and active power from exceeding their limits. The effectiveness of MTDC grid control is further improved through a coordinated control approach^11^, which merges the droop control and voltage margin control based on the fixed power and DC voltage references. Although this research ensures that VSCs operate within their rated capacities, it could cause DC voltage deviation when substantial variations in power occur. To order to address this issue, coordinated control methods have been developed using the droop coefficient, which is derived on the basis of the system stability limit^12^ and a secondary DC voltage regulation technique^13^. The predetermined VSC capacity in these techniques may cause power imbalances and DC voltage instability under severe grid disturbances. Owing to its high reliability, droop control is well suited for applications in a VSC-MTDC grid, allowing the VSC terminal to autonomously regulate power and DC voltage without relying ona fast communication linkage^14,15^. Consequently, fixed droop control approaches have been designed, where adjust droop coefficient is adjusted to optimize power sharing and voltage regulation within MTDC network^16^. Since these studies operate VSCs strictly under the rated capacities, a substantial power imbalance can still lead to DC voltage variations. Recent research^17,18^ has incorporated a virtual synchronous generator into variable droop control (VDC) to enhance MTDC grid performance. These studies consider only the rated capacity of power without accounting for available VSC headroom during operation. The VDC decentralizes the voltage regulation among multiple VSC stations but it inherently causes DC voltage deviation due to imbalanced power allocation during large power imbalances in the MTDC^19^. Thereby, several VDC strategies have been developed that dynamically adjust droop coefficients based on the headroom of each VSC to enhance overall control performance, as presented in^20,21^. These methods still fail to enforce strict DC voltage and active power limits, allowing them to surpass their operational limits under major power disruptions.

The concept of converter headroom is applied in^8^ to develop the VDC aimed at improving control performance, but an outage of the master VSC produces unequal power management and causes perturbation in the DC voltage within the grid. In^22^, authors have proposed VDC focusing on power sharing, but these control schemes do not adequately address the stabilization of the DC voltage profile within MTDC grid. While research in^23,24^ has examined how the droop constant on rectifier VSC affects power balance and voltage stability during disturbance. These studies do not consider the effects on the performance of inverting VSC under significant power disturbance conditions. Additionally, the VDC scheme utilizes a dynamic loading factor derived from the converter headroom for improving MTDC operation^25^. This method neglects the relationship between regulation of balanced power between rectifying and inverting VSC and DC voltage. Furthermore, the VSC headroom on the rectifier side is utilized to develop the VDC approach, boosting control effectiveness in the AC-MTDC system^26^. This scheme depends on the communication network, as a fault can lead to unbalanced power allocation and DC voltage instability issues. Proportional integral and lead lag controllers have been used in^27^ to improve control performance in MTDC. However, this control approach is designed without evaluating its influence on the active power in the grid. In^28^, authors have examined the effect of voltage fluctuations on HVDC system based on VSC. A power planning methodology that addresses the coordinated development of both power production and transmission network, considering the optimal placement and generation unit sizing, and HVDC system is provided in^29^. These analyses are carried out without considering the DC voltage and active power regulation parameters. The summary of the literature review is shown in Table 1.

**Table 1:** Summary of the literature review.

Ref	Control method	Headroom is used on VSC of		Parameter within safe limits		Verified the stability of the system	Performance under various operating modes		year
		rectifier	Inverter	DC voltage	Power		Normal	Disturbance	
^8^	VDC	$\checkmark$	$\times$	$\checkmark$	$\checkmark$	$\times$	high	medium	2019
^9^	Hierarchical Control	$\times$	$\times$	$\checkmark$	$\times$	$\checkmark$	high	low	2025
^10^	Model predictive control	$\times$	$\times$	$\times$	$\checkmark$	$\times$	low	$\times$	2025
^11^	coordinated control	$\times$	$\times$	$\checkmark$	$\checkmark$	$\checkmark$	high	low	2023
^12^	coordinated control	$\times$	$\times$	$\checkmark$	$\checkmark$	$\checkmark$	high	low	2021
^13^	coordinated control	$\times$	$\times$	$\checkmark$	$\times$	$\checkmark$	high	low	2023
^14^	Droop control	$\times$	$\times$	$\checkmark$	$\checkmark$	$\times$	Low	$\times$	2025
^15^	Droop control	$\times$	$\times$	$\checkmark$	$\checkmark$	$\times$	high	low	2025
^16^	Droop control	$\times$	$\times$	$\checkmark$	$\checkmark$	$\times$	low	$\times$	2023
^17^	Virtual Synchronous Generator	$\times$	$\times$	$\checkmark$	$\checkmark$	$\checkmark$	low	$\times$	2025
^18^	Voltage droop control	$\times$	$\times$	$\checkmark$	$\checkmark$	$\checkmark$	low	$\times$	2025
^19^	Voltage droop control	$\times$	$\times$	$\checkmark$	$\checkmark$	$\checkmark$	high	low	2020
^20^	VDC	$\checkmark$	$\times$	$\checkmark$	$\checkmark$	$\checkmark$	low	$\times$	2022
^21^	VDC	$\checkmark$	$\times$	$\checkmark$	$\checkmark$	$\times$	high	low	2019
^22^	Droop control	$\times$	$\times$	$\checkmark$	$\checkmark$	$\times$	low	$\times$	2019
^23^	VDC	$\checkmark$	$\times$	$\checkmark$	$\checkmark$	$\checkmark$	high	medium	2023
^24^	Variable droop control	$\times$	$\times$	$\checkmark$	$\times$	$\times$	low	$\times$	2019
^25^	VDC	$\checkmark$	$\times$	$\checkmark$	$\checkmark$	$\times$	low	$\times$	2021
^26^	VDC	$\checkmark$	$\times$	$\checkmark$	$\checkmark$	$\checkmark$	high	low	2024
^27^	Improved proportional control	$\times$	$\times$	$\checkmark$	$\times$	$\times$	low	$\times$	2025
Our paper	HR-ADC	$\checkmark$	$\checkmark$	$\checkmark$	$\checkmark$	$\checkmark$	high	high	Cont..

### Research gaps and contributions

The literature review reveals that existing droop control approaches in VSC-MTDC grid often rely on the rated capacities of VSC. These methods can overburden converters with small power rating under large disturbances, leading to excessive DC voltage fluctuations and unequal power allocation in MTDC. Moreover, the VSC headroom has been incorporated into the droop coefficient without considering the effect of inverting VSCs on DC voltage stability and active power distribution within MTDC during severe power disturbances. Furthermore, control schemes are introduced without analyzing their effect on overall system stability. To overcome these drawbacks, this research paper proposes the VSC headroom based adaptive droop control (HR-ADC) approach that adaptively varies the droop coefficient to enhance DC voltage regulation and power sharing in the case of large power instabilities in MTDC. Unlike VDC methods, the designed HR-ADC employs the droop coefficient derived from the headroom of inverter and rectifier converters to optimize power redistribution. This approach ensures improved system stability under large power imbalances. The key contributions of this work are as follows:

1. The HR-ADC scheme is suggested to optimize active power management and DC voltage within MTDC network using the headroom of the rectifier and inverter VSCs.
2. The proposed droop coefficient is developed according to the headroom of rectifier and inverter VSCs to dynamically tune the operating conditions of converter terminals, thereby improving DC voltage regulation and ensuring effective power sharing during major grid disturbances. Additionally, root locus analysis is used to validate the impact of the designed droop coefficient on the stability of the MTDC network.
3. The proposed HR-ADC is tested under extreme conditions of operation, such as VSC outages and fault scenarios. Its superior performance is also confirmed through comparison with the VDC method.

### Paper organization

The remaining parts of this paper are structured as follows: Section 2 describes the DC voltage and power relationship within MTDC. Section 3 includes the VDC. Section 4 presents the designed HR-ADC method. Section 5 presents the simulation outcomes, and Section 6 outlines the conclusion.

## DC voltage and active power relationship in MTDC

The basic droop control principle serves as a standard approach for evaluating operating conditions in MTDC system. This method ensures proper active power distribution to stable DC voltage stability, as expressed by the voltage and power relationship in Eq. 1.

1 $$\begin{aligned} V_{dcref}-V_{dc} =k_{droop}(P-P_{ref}) \end{aligned}$$

where, $P_{ref}$ and $V_{dcref}$ are the references of active power and DC voltage, $k_{droop}$ is the droop coefficient of the converter terminal. The control characteristic can be further simplified as follows

2 $$\begin{aligned} \Delta P= & (P-P_{ref}) \end{aligned}$$

3 $$\begin{aligned} \Delta V_{dc}= & V_{dcref}-V_{dc} \end{aligned}$$

4 $$\begin{aligned} \Delta V_{dc}= & k_{droop}\Delta P \end{aligned}$$

For power disturbance $\Delta V$, the power deviation of the ith VSC

5 $$\begin{aligned} \Delta P= & \sum _{n}^{i=1}\Delta {P_{i}}=\Delta V_{dc}\sum _{n}^{i=1}\frac{1}{k_{droopi}} \end{aligned}$$

6 $$\begin{aligned} \Delta P= & {k_{droopi}} \Delta {P_{i}}\sum _{n}^{i=1}\frac{1}{k_{droopi}} \end{aligned}$$

7 $$\begin{aligned} \Delta P_{n}= & \frac{\Delta P}{{k_{droopn}}}\sum _{n}^{i=1}\frac{1}{k_{droopn}} \end{aligned}$$

The droop coefficient $(k_{droopi})$ in DC voltage control, as expressed in Eq. 7, determines how imbalanced power is regulated among VSCs in an MTDC system. If all VSC terminals share identical $(k_{droopi})$ the imbalanced power is distributed equally. However, when $k_{droopi}$ differs, VSC with larger $k_{droopi}$ contributes less to balancing power, while a VSC with smaller $k_{droopi}$ compensates for a greater share of the power imbalance.

## Variable droop control (VDC)

**Fig. 1:**
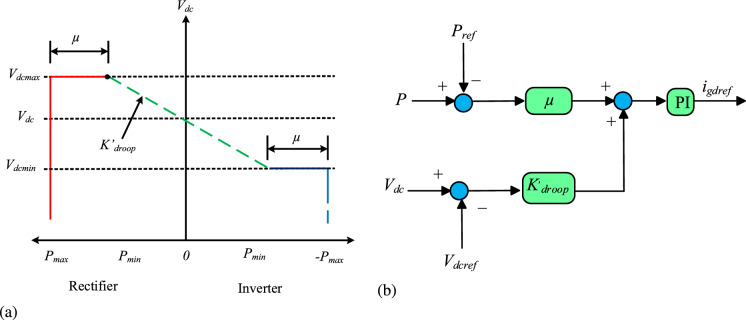
Conventional VDC (**a**) voltage and power characteristic, and (**b**)*d*q control schematic diagram.

Fixed droop control allocates power based on the rated capacity of VSCs, which leads to imbalanced power distribution and unstable DC voltage fluctuations within MTDC, especially under major power disturbances. To address these limitations, VDC has been introduced, as explored in^13–15,20,26^. These methods determine the droop coefficient by considering the available headroom of each VSC, using predefined power values, as illustrated in Eq. 8. The VDC consists of *d*q diagram, and the voltage and power characteristic curve is given in Fig 1.

8 $$\begin{aligned} k'_{\text {droop}} = {\left\{ \begin{array}{ll} \frac{\mu (P_{\text {max}} - P)}{P_{\text {max}}} \times k_{\text {droop}} & \text V_{\text {dc}} > V_{\text {dcref}} \\ \frac{\mu P}{P_{\text {max}}} \times k_{\text {droop}} & \text V_{\text {dc}} \le V_{\text {dcref}} \end{array}\right. } \end{aligned}$$

where *P* and $P_{\max }$ are the measured power and maximum power of VSC, respectively. $\mu$ is the headroom of the converter, and $k'_{droop}$ represents the droop coefficient for the VDC method. In an MTDC grid employing VDC, the slope of voltage and power (V-P) exhibits notable variations in power output and DC voltage due to changes in power and voltage values. As per Eq. 8, $k'_{droop}$ is determined by the available headroom of the converter based on the maximum and minimum power and DC voltage values and directly affects power allocation. A higher value of $k'_{droop}$ at a VSC results in a smaller power margin but a much larger power distribution. Similarly, a lower droop coefficient value increases the power margin values, reducing the power share at the VSC station. However, under the large power disturbances, VSCs with minimum power values risk overloading, while those with large power values operate near their rated capacity. This imbalance leads to improper power regulation and considerable fluctuations in DC voltage throughout the MTDC.

It should be emphasized that the term VDC refers to a broad family of control schemes in MTDC system, including variants that incorporate adaptive droop gain, headroom considerations, and predefined power rating of VSCs. In this study, the modified droop law for VDC is adopted as the baseline given in Eq. 8. It represents the widely implemented VDC method based on voltage and power characteristics in the existing MTDC systems, which provides a clear and well defined reference for comparison. As a result, Eq. 8 is considered a representative benchmark for evaluating the improvements introduced by the proposed HR-ADC. Furthermore, the comparative performance is evaluated using the peak DC voltage deviation and power sharing parameters under the large power disturbance to ensure a fair and transparent assessment.

## Proposed headroom based adaptive droop control scheme

**Fig. 2:**
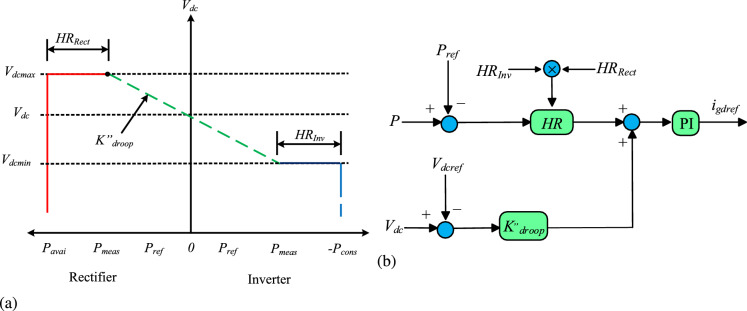
Proposed HR-ADC (**a**) voltage and power characteristic, and (**b**) *d*q control schematic diagram.

The VDC technique is widely used to control DC voltage and manage active power. During large power imbalance scenarios, the VSC in VDC assists in stabilizing the grid by sharing the load power to ensure reliable and stable system performance. However, the use of the conventional droop coefficient prevents the VSC from restricting its power contribution within its rated capacity. As a result, the VDC may experience overloading, thereby compromising the stability of DC voltage. To address this issue, the contribution of the VSC shown in Fig 1 is enhanced as illustrated in Fig 2. In this design, the proposed droop coefficient for HR-ADC is calculated by employing the converter headroom of both rectifying and inverting converters, as shown in Eq. 9 and Eq. 10, respectively.

9 $$\begin{aligned} k_{\text{ droop } }^{\prime \prime }= & \left\{ \begin{array}{cc} \frac{P_{\text{ avail } }- P_{\text{ meas } }}{P_{\text{ max } }} \times k_{\text{ droop } } & V_{d c}>V_{\text{ dcref } } \\ \\ \frac{P_{\text{ meas } }-P_{\text{ ref } }}{P_{\text{ max } }} \times k_{\text{ droop } } & V_{d c} \le V_{\text{ dcref } } \end{array}\right. \end{aligned}$$

10 $$\begin{aligned} k_{\text{ droop } }^{\prime \prime }= & \left\{ \begin{array}{cl} \frac{P_{\text{ consume- } } P_{\text{ meas } }}{P_{\text{ max } }} \times k_{\text{ droop } } & V_{d c}>V_{\text{ dcref } } \\ \\ \frac{P_{\text{ meas } }-P_{\text{ ref } }}{P_{\text{ max } }} \times k_{\text{ droop } } & V_{d c} \le V_{\text{ dcref } } \end{array}\right. \end{aligned}$$

where $P_{avail}$, $P_{meas}$, and $P_{cons}$ are the available power supply from RES, the measured power capacity of converter terminals, and power used by the inverting converters. $k^{,,}_{droop}$ is the designed droop coefficient of HR-ADC. The designed HR-ADC scheme addresses these challenges by enhancing the stable and reliable operation of MTDC grid, even under intense power imbalances conditions. This is accomplished by efficiently allocating active power and regulating DC voltage through the utilization of the developed droop coefficient.

In the developed droop coefficient, inverting converter headroom (${HR_{Inv}}$) is maintained by operating within the range defined by its maximum power consumption and actual measured values, as defined in Eq. 11. ${HR_{Inv}}$ is determined by the difference between maximum power utilization and measured value. Essentially, ${HR_{Inv}}$ represents the margin between its rated capacity and the actual power it is consuming. In contrast, rectifying converter headroom (${HR_{Rect}}$) is determined by the difference between the maximum available power and the rated measured value, as provided in Eq. 12. $P_{avail}$ at the rectifying converter is estimated or predicted as it fluctuates over time. As in this study RE based power generation is used at the rectifier converter. As a result, converter headroom plays an important role in enhancing active power balance and stabilizing DC voltage.

11 $$\begin{aligned} HR_{Rect}= & P_{avail}- P_{meas} \end{aligned}$$

12 $$\begin{aligned} HR_{Inv}= & P_{cons}- P_{meas} \end{aligned}$$

The proposed $K^{,,}_{droop}$ is given in Eq. 9 and Eq. 10 works by adjusting its value based on the available headroom of each VSC (${HR_{Rect}}$ and ${HR_{Inv}}$) which directly impacts the power balance and DC voltage. The values of ${HR_{Rect}}$ and ${HR_{Inv}}$ are provided in Eq. 11 and Eq. 12, respectively. When $K^{,,}_{droop}$ is set to a higher value, the available power margin at ${HR_{Rect}}$ and ${HR_{Inv}}$ becomes smaller, while the assigned power increases. In this situation, ${HR_{Rect}}$ and ${HR_{Inv}}$ operates proportionally to their remaining capacity of headroom. This causes the VSC to reduce its power more aggressively, thereby preventing the VSC from overloading in MTDC system. In contrast, with lower $K^{,,}_{droop}$ values, the power margin at the ${HR_{Rect}}$ and ${HR_{Inv}}$ remains large, leading to a reduce droop slope, which helps to maintain the stable DC voltage level and regulated the balance power in the MTDC system. This allows the MTDC system to achieve stable DC voltage control and balance power allocation under changing operating conditions, which cannot be achieved using the VDC scheme.

### Control structure and operating principle

**Fig. 3:**
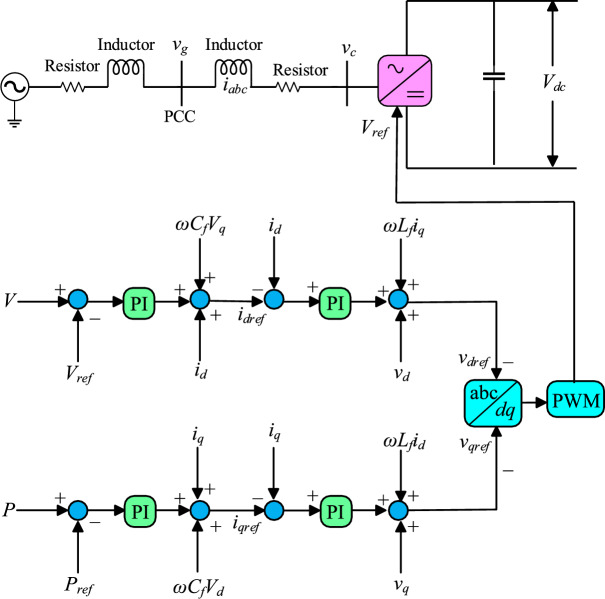
dq control structure of the proposed HR-ADC system.

**Fig. 4:**
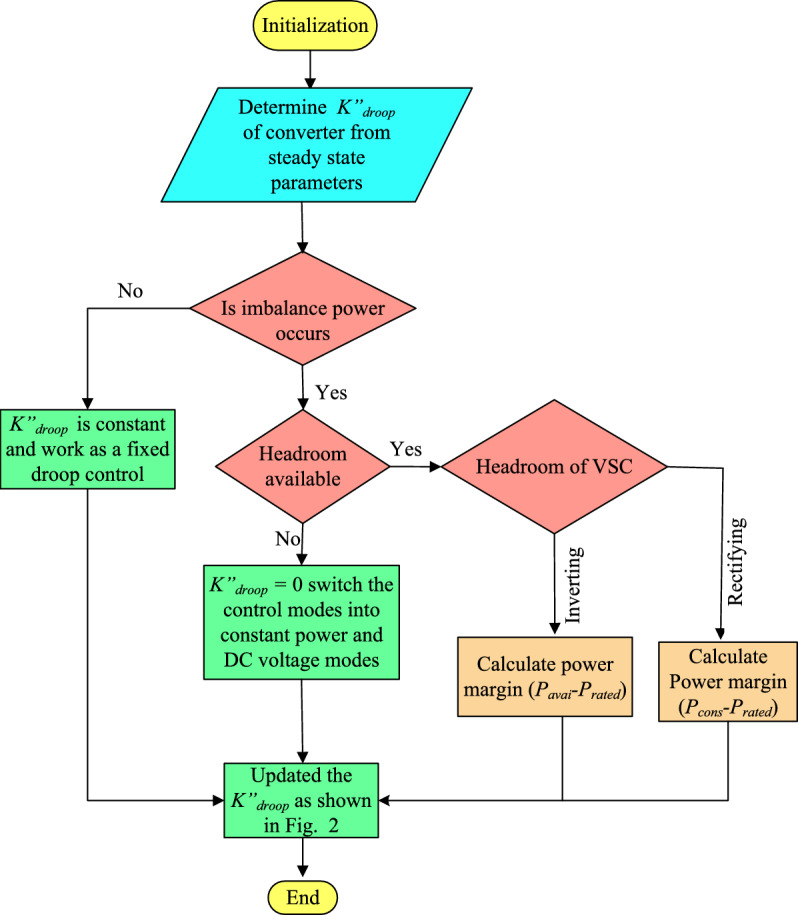
Flow chart of an HR-ADC scheme.

This study uses the *d*q-axis control mechanism discussed in^8^ but with a modification in the VSC headroom in the droop coefficient design, as illustrated in Fig 3. Fig 3 presents a control structure of the VSC-HVDC terminal that is composed of inner and outer control loops. The inner control loop controls a synchronously rotating *d*q reference frame which is voltage oriented, and the grid currents ($i_d$ and $i_q$) are controlled to follow their reference values ($i_{dref}$ and $i_{qref}$) rapidly by regulating the output voltage of the VSC. As a result, the inner current has direct control over the converter current and power exchange, depending on the outer loop. To ensure decoupled control in the dq frame, the loops are designed with compensation terms ($\omega L_qi_d$, and $\omega L_qi_q$) and feedforward compensation based on AC system voltages ($v_q$ and $v_d$). The detailed structure of the inner current loop controller is provided in Fig 3. The outer controller controls the active power of the VSC and DC voltage and produces reference currents to the inner current loop. Based on operating modes, the reference for the active current $i_{qref}$ is determined by constant power or voltage, and the droop controller. Under normal working conditions, the signal applied to *P*I control is zero. Therefore, Eq. 13 establishes the link between DC voltage and power.

13 $$\begin{aligned} V_{dcref}-V_{dcmeas} =K^{\,,,}_{droopi}(P_{ref}-P_{meas}) \end{aligned}$$

where, $P_{ref}$ and $V_{dcref}$ are reference values for power and DC voltage, $P_{meas}$ and $V_{dcmeas}$ are the measured values for power and DC voltage. The parameter $K^{,,}_{droop}$ represents the designed droop coefficient, which is determined based on the power margin of rectifying or inverting VSC terminals, as specified in Eq. 9 and Eq. 10, respectively. Adjustment of the $k^{,,}_{droop}$ value leads to a shift in the *d*q reference frame within the control loops, as shown in Fig 4. This conversion enables the regulation of active power and DC voltage, supporting the transition between different control modes. The values of $k^{,,}_{droop}$ fluctuate according to the headroom of rectifying and inverting VSCs as explained in Eq. 11 and Eq. 12, respectively. This variation leads to modifications in the control modes of the MTDC and strengthen system stability during significant power disruptions. As a result, ensuring HR-ADC has variable behavior. This HR-ADC approach enhances DC voltage and power regulations in MTDC by varying the $k^{,,}_{droop}$ and operating within the VSC’s headroom limits. Unlike conventional modes of operation that rely on fixed set points for DC voltage and active power ($P_{ref}$ and $V_{dcref}$), HR-ADC responds dynamically to imbalance power conditions. Moreover, the control strategy is quite reliant on the local situation and uses parameters that are measured independently at every VSC. Therefore, the HR-ADC operates without relying on data from other VSCs, eliminating the need for a communication link within the control structure. The procedure illustrated in Fig 4 can be followed to compute the designed droop coefficient.

In the flow chart of HR-ADC, the control framework is adapted from^23^ with modified control parameters. By following the flowchart, the $k^{,,}_{droop}$ operates effectively under normal MTDC grid conditions. Based on the droop coefficients evaluation, the HR-ADC dynamically adjusts the control modes within MTDC grid. In this mode power sharing among VSCs remains balanced, and no headroom is required for system operation. As a result, $k^{,,}_{droop}$ functions similarly to a fixed droop control under constant voltage and power regulation modes. However, during major power disturbances, including VSC outage or fault scenarios, the VSC terminal with a rated capacity of power may experience overloading. To address this problem, the HR-ADC adjusts the $k^{,,}_{droop}$ by changing the rectifying VSCs to droop voltage mode, while the inverting VSCs are altered droop power mode. This adjustment helps maintain balanced power distribution and stabilizes the DC voltage within the MTDC grid. When the conditions stabilize within the system, the VSCs revert to their standard operating modes, where power is distributing power based on their rated capacities. The corresponding $k^{,,}_{droop}$ values are updated as given in Fig 4.

The proposed droop coefficient automatically shifts the converter stations into droop control mode when the DC voltage deviates the reference value due to a fault. This transition helps mitigate large DC voltage variations and supports balanced power sharing. The switching of the modes of operation takes place within available headroom of the VSC, leading to a change in the *dq*-axis current reference within the control loop, as shown in Fig 2b. These adjustments lead to changes in DC voltage and power, guiding the system to reach a new steady state operating condition. Accordingly, during a significant power interruption, the designed headroom for VSCs is employed to avoid overloading, stabilize the DC voltage, and ensure balanced power allocation among VSCs within the MTDC grid. In other words, to provide stable DC voltage and maintain balanced power in MTDC, the proposed HR-ADC dynamically modifies the output power of each VSC through $K^{,,}_{droop}$, which adjusts to the available headroom of rectifying and inverting VSCs. When the DC voltage deviates from its rated value, the HR-ADC mechanism reallocates power among VSC proportionally to their remaining capacity and switches operational modes to either droop voltage or droop power. This prevents excessive voltage fluctuations and overloading of the small power converter. This coordinated power adjustment supports DC voltage and power balancing across the entire MTDC system. Moreover, the inherent decentralized structure allows each VSC to respond rapidly using only local measurements, which enhances overall stability under normal operations and disturbances.

### Stability analysis of proposed HR-ADC

**Fig. 5:**
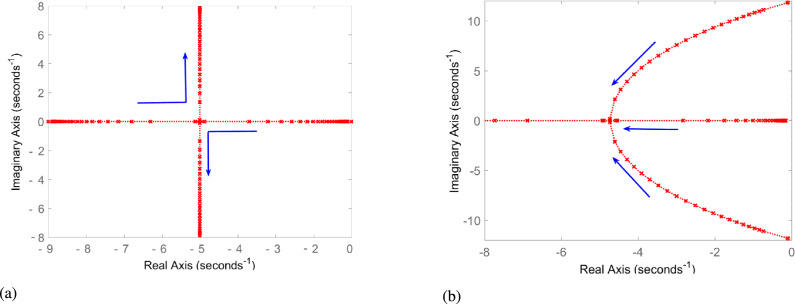
Root locus of the network under varying values of $K^{,,}_{droop}$ (*a*) normal operating mode, (*b*) large power disturbance operating modes.

This study employs the *dq* frame structure control presented in^8^. The transfer function for the developed HR-ADC is derived from this reference control framework and is provided below Eqs.

14 $$\begin{aligned} G_{inner} (s)= & \frac{K_p(s)}{L_{eq}(s)^2+R_{eq}(s)} \end{aligned}$$

15 $$\begin{aligned} Q_{inner}(s)= & \frac{K_p(s+1)G_{inner} (s)}{k_d G_{inner}(s)+T_d(s+1)} \end{aligned}$$

16 $$\begin{aligned} G(s)= & \frac{k_p(s)}{1-k^{,,}_{droop}(k_p)Q_{inner}(s)} \end{aligned}$$

17 $$\begin{aligned} Q= & \frac{G(s)}{1+G(s)} \end{aligned}$$

where, the term $G_{inner} (s)$ represents the inner loop transfer function, while $K_p$ denotes the proportional gain, and $K^{,,}_{droop}$ is the droop coefficient. $L_{eq}$ and $R_{eq}$ are the equivalent inductance and resistance, respectively, and $Q_{inner}(s)$ refers to the outer loop transfer function. The term $K_p(s+1)$ indicates proportional gain with a shift by 1 in the domain of Laplace. $K_d$ is the derivative gain constant, and $T_d(s+1)$ indicates the time constant derivative expressed in terms of the Laplace variable *S*. *G*(*s*) is the system gain, and *Q* present the closed loop transfer function. The $K^{,,}_{droop}$ is a critical parameter in the proposed HR-ADC method. Root locus assessment is employed to evaluate the impact of $K^{,,}_{droop}$ on the DC voltage stability. The root locus of the MTDC system is given in Fig 5a and Fig 5b, respectively, representing the normal and huge power disturbance scenarios, with blue arrows indicating the shifts resulting from variations in the $k^{,,}_{droopi}$.

In the normal operating mode, root loci lie in the left half area of the s plan, demonstrating that the HR-ADC maintain system stability. Under this scenario, $k^{,,}_{droop}$ is either fixed or changes uniformly without affecting system stability. During significant power interruptions, $k^{,,}_{droop}$ increases, and all loci remain in the left half of the s plane, signifying stable operation of the developed HR-ADC as provided in Fig 5b. The structure of the root locus varies as the value of $k^{,,}_{droop}$ changes; however, the system remains stable. The data indicates that as $k^{,,}_{droop}$ increases, the root loci remain clustered in the left half area in s plan, confirming that the network is operates in a stable state. In this HR-ADC, the droop coefficient plays a significant role in enhancing the DC voltage stability. By increasing the droop coefficient, overall stability in the network is continuously improved.

## Simulation and results

**Fig. 6:**
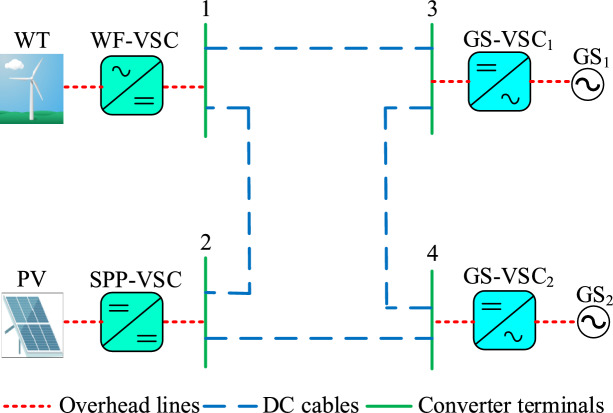
The MTDC test system.

A four terminal VSC based MTDC system operating at ± 400 kV was developed in PSCAD to evaluate the performance and effectiveness of the designed HR-ADC in mitigating DC voltage fluctuations and managing power regulation, as provided in Fig 6. The test system includes VSCs connected to solar power plant (SPP), a wind farms (WF) and two grid side converters ($\hbox {GS}_1$ and $\hbox {GS}_2$). In this configuration, the MTDC network receives active power from the VSCs of the SPP and WF and distributes it through $\hbox {GS}_1$ and $\hbox {GS}_2$ converter terminals. Additionally, active power may also be imported from the grid and delivered to the connected AC system. Various scenarios, including VSC terminal outages and faults at VSC stations are considered to demonstrate the effectiveness of the HR-ADC strategy.

Table 2 and Table 3 present the designed headroom values for the VSCs and cable line specification^30^ of the planned test system, respectively. In Table 2, the 200 MW headroom allocated to each VSC station is selected as the assumed design power margin, approximately 25 % of each VSC rating. This power margin reflects the typical power limits applied in MTDC system to prevent VSC overload risk under severe disturbances. The rectifier headroom depends on the estimated available power ($P_{avail}$); from the upstream of the RES. The predication errors may impact the adaptation speed rather than the decentralized control behavior of the developed HR-ADC scheme. Although detailed forecasting modeling of PV and WT is not included in this research.

In the HR-ADC scheme, when wind speed is low and solar irradiation is minimal, the SPP and WF converters operate at their rated capacities or predefined setpoints within the MTDC system. However, during periods of high wind and strong solar irradiation, the VSCs associated with WF and SPP operate within the designed headroom of VSC. Conditions with negligible solar input and low wind speed are treated as equivalent to VSC outages. Similarly, under normal scenarios, the GS converter is working at its rated capacities. However, during power disturbances, the GS VSCs utilize their converter headroom to improve control performance.

**Table 2:** The designed headroom of VSCs.

Converters terminal	Rating (MW)	Pre-set value (MW)	Headroom (MW)
GS-VSC1	800	600	200
GS-VSC2	600	400	200
WF-VSC	800	600	200
SPP-VSC	600	400	200

This study does not include detailed modeling and analysis of wind turbines and solar photovoltaic systems. Additionally, the influence of HR-ADC on system frequency is not considered in this work. Since that VDC is commonly used in MTDC systems to improve control performance, the effectiveness of the designed HR-ADC is assessed by comparing its simulation results with a benchmark VDC implementation.

**Table 3:** Cable line data.

Parameters	Rating
DC voltage	400 kV
Maximum DC voltage	420 kV
Minimum DC voltage	380 kV
Minimum DC voltage	380 kV
Resistance of the line	0.0095 $\Omega$/km
Inductance of the line	2.110 mH/km
Capacitance of the line	0.1906 $\upmu$F
Frequency of the system	50 Hz
L12 to VSC line length	50 km
L34 to VSC line length	75 km
L24 to VSC line length	75 km
L13 to VSC line length	50 km

### Validation and comparative analysis of the proposed HR-ADC with the VDC

**Fig. 7:**
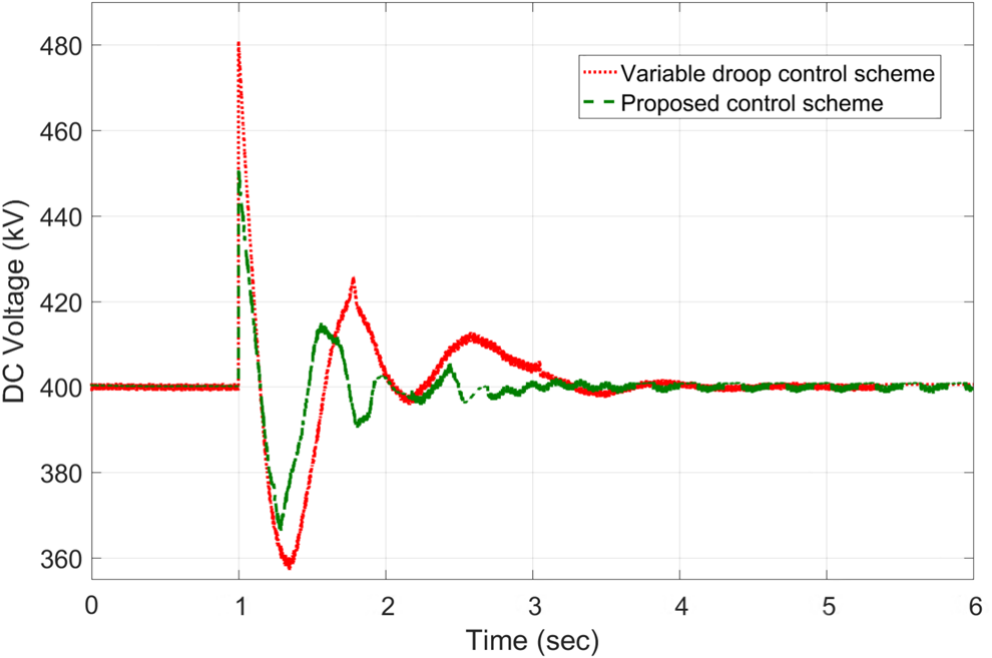
DC voltage performance response in MTDC grid.

**Fig. 8:**
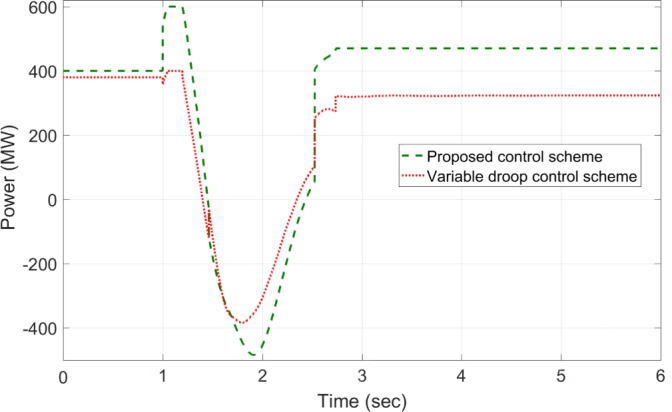
Response of power regulation in MTDC grid.

The response of sustaining DC voltage and ensuring power regulation within MTDC using the VDC and the designed HR-ADC are shown in Fig 7 and Fig 8.

Initially, at t= 0 to 1 second (s), VSCs of VDC and HR-ADC supply constant power of 380 and 400 MW to the network in fixed power mode, respectively. At t= 1 s, the VDC transitions to droop control based on the droop coefficient defined in Eq. 8, increasing its power injection to 400 MW to the system and reaching the highest specified capacity of the designed fixed power VSC as given in Table 2. This causes the VSC using VDC to become overloaded, resulting in a DC voltage rise to 480 kV as given in Fig 7. In contrast, under the same condition, the HR-ADC responds by shifting from fixed power mode to droop power mode, using the proposed droop coefficient value as discussed in Eq. 9 and Eq. 10.

The HR-ADC imports 600 MW into the grid, effectively utilizing the converter’s headroom as specified in Table 2. Therefore, the converter terminal operates within the proposed headroom of the power converter, maintaining a DC voltage of 420 kV in the grid. At t = 2 s, the HR-ADC absorbs -483 MW from the system, contributing to an improvement in DC voltage stability. In comparison, MTDC under VDC exports -354 MW, leading to DC voltage exceeding the stability threshold. After t = 3 s, the designed HR-ADC converter supplies 470 MW to the network by shifting the operational mode from variable power to uniform power. Meanwhile, the VSC under ADC imports 322 MW into the MTDC, which causes a shift in the operational modes to fixed active power. Between t=3 to 6 s, both VDC and HR-ADC operate in fixed power modes, supplying uniform power to the grid to stabilize DC voltage and regulate power.

The simulation outcome illustrates that the HR-ADC enhances the effectiveness of the current VDC in regulating active power and DC voltage within MTDC grid.

### Performance analysis of the HR-ADC and VDC under the condition of converter outage

The response of DC voltage and power regulations under converter outage operating scenarios is conducted using VDC and planned HR-ADC schemes. This assessment aims to highlight the control effectiveness and demonstrate the superiority of the designed HR-ADC within MTDC grid. The following VSC outage conditions are considered in the analysis.

#### Case 1: SPP out of service

In this case, during the initial interval from t = 0 to 0.9 s, WF and SPP converters import 523 and 398 MW power respectively into MTDC while operating in uniform voltage mode. Simultaneously, the grid side ($\hbox {GS}_1$, $\hbox {GS}_2$) VSCs respectively export -371 and -550 MW power in uniform power mode. As a result, VDC and the proposed HR-ADC maintain fixed power and voltage operation, ensuring equal power management and stable DC voltage across the grid. Following the outage of SPP-VSC, the output power of the SPP converter rapidly drops to zero from t=1 to 3 s.

**Fig. 9:**
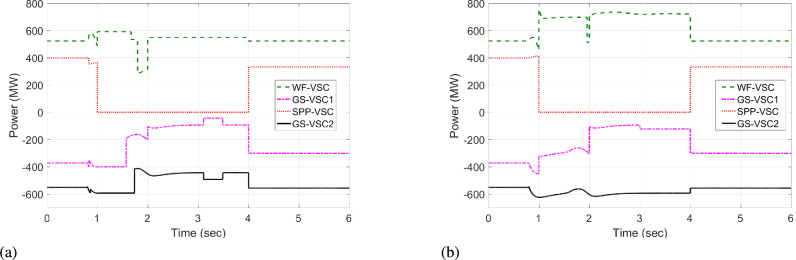
Response of power regulation in case 1; (**a**) VDC, and (**b**) HR-ADC.

**Fig. 10:**
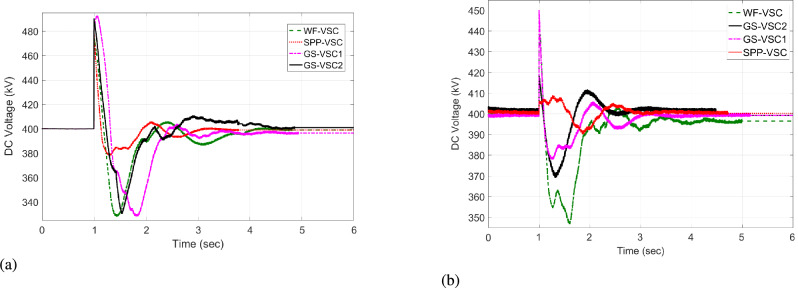
DC voltage response in case 1; (**a**) VDC, and (**b**) HR-ADC.

The VSC stations of WF, $\hbox {GS}_1$, and $\hbox {GS}_2$ then utilize the unequal power in the network. Under this scenario, the VDC operates according to the droop coefficient value as discussed in Eq. 8 and utilizes the fixed power values in the grid. Therefore, WF converter delivers 600 MW to the network, whereas grid side VSCs import -222 and -600 MW from the grid and operate at their maximum rated capacities, as illustrated in Fig. 9a. Hence, the VSCs of WF, $\hbox {GS}_1$, and $\hbox {GS}_2$ are overloaded, resulting in 475, 485, and 490 kV DC voltage fluctuations in the grid, as given in Fig 10a. In contrast, the proposed HR-ADC adapts to the VSC outage by utilizing the developed droop coefficient and altering the operational modes of WF, $\hbox {GS}_1$, and $\hbox {GS}_2$ to voltage and power droop to mitigate the DC voltage variation. Under this mode, VSC of WF distributes 755 MW, and GS-VSC2 consumes -622 MW in the grid in the voltage and power droop modes, respectively, which enhances the unequal power utilization, as provided in Fig 9b. This enhanced regulation improves the DC voltage stability in the network with $\hbox {GS}_1$, $\hbox {GS}_2$, and WF converter registering voltage level of 439, 429, and 424 kV, respectively, as displayed in Fig 10b. These VSCs dynamically adjust their operational modes according to the values of the designed droop coefficient of the rectifying converter, as provided in Eq. 9. Despite minor voltage fluctuations in DC voltage at the WF terminal, the system restore sustains balanced power regulation and effectively keeps DC voltage after t = 2 s.

The power of SPP is restored at t = 4 s. The converter stations of WF and SPP transfer 523 and 333 MW to the MTDC network, and converter stations $\hbox {GS}_1$ and $\hbox {GS}_2$ consume -301 and -556 MW from the grid at t = 4 to 6 s. As a result, both VDC and HR-ADC controllers maintain power-sharing between the rectifying and inverting converter stations, resulting DC voltage within the stability boundary. Therefore, DC voltage variation is stabilized by the $\hbox {GS}_1$, WF, and $\hbox {GS}_2$ converter stations. According to the findings of case 1, VSC stations of WF, $\hbox {GS}_1$ under HR-ADC have distributed the proper power, maintaining DC voltage within MTDC by altering the control mode. The comparison results demonstrate the effectiveness of HR-ADC over VDC in maintaining power balance and voltage regulation.

#### Case 2:WF converter outage

**Fig. 11:**
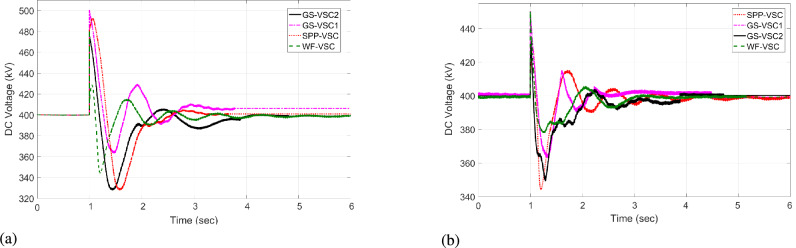
DC voltage response in case 2; (**a**) VDC, and (**b**) HR-ADC.

**Fig. 12:**
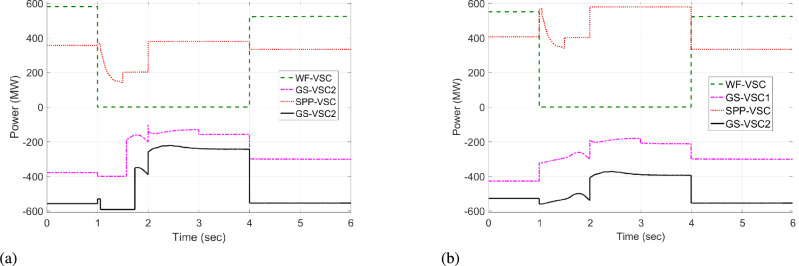
Response of power regulation in case 2; (**a**) VDC, and (**b**) HR-ADC.

In case 2, under steady state period from t = 0 - 0.9 s, and t = 4 - 6 s, both HR-ADC and VDC techniques operate in the fixed voltage and power states to stable DC voltage and ensure balanced power within MTDC as given in Fig 11 and Fig 12. An outage of the WF converter station occurs at t=1 s, with restoration taking place at t = 4 s. During this disturbance, the SPP-VSC is incapable of participating in the power regulation in MTDC grid. As a result, the responsibility for regulating imbalanced power falls on the VSCs of $\hbox {GS}_2$, SPP, and $\hbox {GS}_1$. Under this scenario, the VDC functions based on the droop coefficient value, as discussed in Eq. 8, and makes use of the rated capacity of power VSC in the system. Therefore, VSCs of SPP contribute 350 MW to the grid. Meanwhile, grid side converters draw -400 and -600 MW from the grid, operating at their maximum rated capacities as illustrated in Fig 12a. Hence, the converter stations of SPP, $\hbox {GS}_1$, and $\hbox {GS}_2$ experience overloading problems, leading to variations in 485, 500, and 490 kV DC voltage in the network, as given in Fig 11a.

The proposed HR-ADC, during the VSC outage scenario, utilizes the variable power margin and changes the operating modes of $\hbox {GS}_2$, SPP, and $\hbox {GS}_1$ to the power and voltage droop modes to stable the DC voltage variation. In this operational mode, the VSC of SPP distributes 350 MW, while $\hbox {GS-VSC}_1$ and $\hbox {GS-VSC}_2$ consume -300 and -500 MW in power droop modes within the grid. This leads to uneven power utilization, as shown in Fig 12b. The power regulation contributes to an enhancement in DC voltage with MTDC, resulting in deviations of 445, 430, and 440 kV DC voltage for the VSCs of SPP, $\hbox {GS}_2$, and $\hbox {GS}_1$ in the MTDC grid as displayed in Fig 11b. These VSCs adjust their operational states according to the specified values of the droop coefficient and use the developed power margin of the rectifying VSC, as outlined in Eq. 10. Subsequently, following variable deviations of DC voltage in VSC of SPP, $\hbox {GS}_1$, and , $\hbox {GS}_2$ the system keeps the regulation of balanced power that manages DC voltage by t = 2 s.

As per the result from case 2, VSC stations of SPP, $\hbox {GS}_1$, and $\hbox {GS}_2$ operating under designed HR-ADC successfully shared the appropriate power power and sustained DC voltage within MTDC by altering the control mode. The comparative results indicate that HR-ADC is more effective than VDC.

#### Case 3: $\hbox {GS}_1$ out of operation

**Fig. 13:**
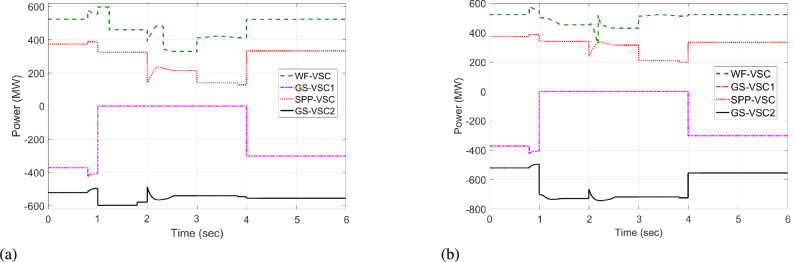
Response of power regulation in case 3; (**a**) VDC, and (**b**) HR-ADC.

**Fig. 14:**
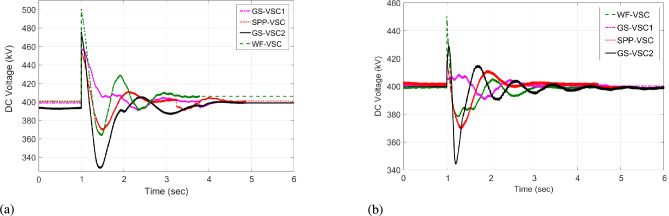
DC voltage response in case 3; (**a**) VDC, and (**b**) HR-ADC.

In case 3, at an initial time from 0 to 0.9 s, VDC, and HR-ADC ensure the power distribution and sustain the DC voltage under normal operational modes. When the $\hbox {GS}_1$ VSC experiences an outage from t=1 to 3 s, it become unbale to participate in the power regulations in the grid. As a result, the unbalanced power is handled by the VSCs of WF, $\hbox {GS}_2$, and SPP. During this condition, VDC operates according to predefined power values of the VSC as given in Eq. 8. Consequently, the VSC of $\hbox {GS}_2$ consume - 598 MW while WF provide 592 MW in the network, as given in Fig 13a. This loading condition leads to DC voltage levels of 500, 450, and 478 kV at the WF, $\hbox {GS}_1$, and $\hbox {GS}_2$, respectively, as displayed in Fig 14a. In compassion to VDC, the presented HR-ADC utilizes the droop coefficient of the inverting converter, as given in Eq. 10. Therefore, $\hbox {GS-VSC}_2$ consumes - 700 MW from the system and alters the control into droop power to avoid VSC overloading problem and improve power-sharing within MTDC, as illustrated in Fig 13b. Hence, the $\hbox {GS}_1$, WF, and $\hbox {GS}_2$ VSCs generate 420, 430, and 450 kV variation in DC voltage in the grid as shown in Fig 14b. After, experiencing some flexible change in the DC voltage of VSC of $\hbox {GS}_2$, the system keeps the regulation of balanced power that manages DC voltage at time 2 s.

At t= 4 s, power is restored, and both control schemes enhance the power allocation DC voltage within MTDC by switching operational states to fixed power and voltage modes, thereby achieving the intended aims. The results from case 3 prove that the HR-ADC enables the VSC stations of the SPP, WF, and $\hbox {GS}_1$ to sustain the active power allocation and stabilized the variation of DC voltage in the network. Overall, the planned HR-ADC outperforms the VDC method.

#### Case 4: $\hbox {GS}_2$ VSC outage

**Fig. 15:**
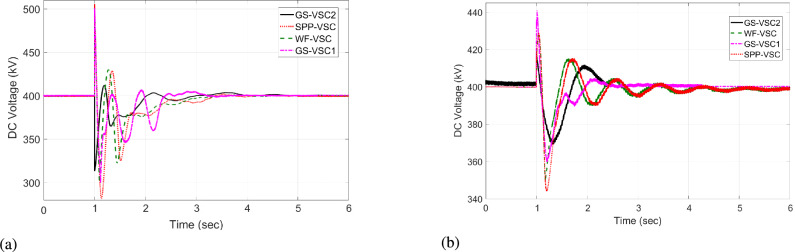
DC voltage response in case 4; (**a**) VDC, and (**b**) HR-ADC.

**Fig. 16:**
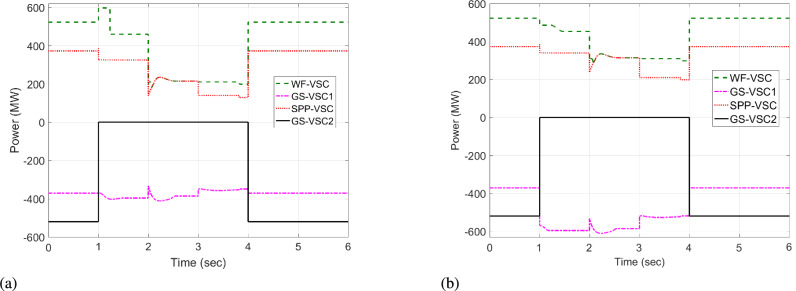
Response of power regulation in case 4; (**a**) VDC, and (**b**) HR-ADC.

In case 4, during the period of t = 0 -1 s and t = 4 - 6 s, both VDC and developed HR-ADC methods function in the fixed voltage and power modes to ensure balanced power distribution and stable DC voltage across the grid. Under this condition, WF and SPP operate in fixed power mode, providing 523 and 373 MW to the system, respectively. Simultaneously, converters of $\hbox {GS}_2$ and $\hbox {GS}_1$ import -525 and -371 MW from the grid in fixed power mode as shown in Fig 15 and Fig 16. Between t=1 to 3 s, $\hbox {GS-VSC}_1$ is unable to contribute in the power allocation within MTDC grid due to the converter terminal outage of $\hbox {GS}_1$. Consequently, the uneven distribution of power is managed by VSCs of WF, $\hbox {GS}_1$, and SPP. During this disturbance, the VDC functions according to the power values of the VSC as given in Eq. 8. Therefore, the VSC of $\hbox {GS}_1$ and WF consume - 598 MW and provide 592 MW to the network, as given in Fig 16a. The power loading of WF-VSC and GS-VSC2 contributes to the generation of the value of DC voltage in the system. Specifically, VSCs of WF, $\hbox {GS}_1$, and SPP produce DC voltages of 500, 450, and 478 kV, respectively, as illustrated in Fig 15a.

Unlike VDC, the presented HR-ADC employs a varying droop coefficient for inverting converters based on predetermined values. This approach enables dynamic adjustment of the operational modes within grid, as specified in Eq. 10. As a result, $\hbox {GS-VSC}_2$ consumes - 700 MW from the grid and alters control mode to the droop to prevent VSC overloading issues, thereby enhancing power-sharing with the MTDC grid, as depicted in Fig 16b. Consequently, VSCs of $\hbox {GS}_1$, WF, and $\hbox {GS}_2$ exhibits DC voltage levels of 420, 430, and 450 kV in the system as shown in Fig 15b. After experiencing fluctuations of DC voltage within VSCs of WF, $\hbox {GS}_1$, and SPP the network successfully upholds balanced power allocation, efficiently controlling DC voltage beyond t = 2 s. Subsequently, after power restoration at t = 4 s, both control schemes improve the power utilization between rectifying and inverting converters and DC voltage within grid by transforming control mode into fixed power and voltage modes, successfully attaining the specified aims.

The results from the $\hbox {GS}_2$ converter outage scenario demonstrate that the HR-ADC implemented in the VSC stations of the SPP, WF, and $\hbox {GS}_1$ maintained power allocation and stabilized DC voltage variation within the grid. The strategically developed HR-ADC surpasses the effectiveness of the VDC.

### Performance analysis of the HR-ADC and VDC methods during the fault at the converter terminal

To evaluate control response and demonstrate the advantages of the designed HR-ADC over the VDC, fault scenarios at the VSCs terminals are analyzed with a focus on the DC voltage control and power allocation. In this study, the following faults at the converter stations are investigated

#### Case I: Fault on the VSC of WF

**Fig. 17:**
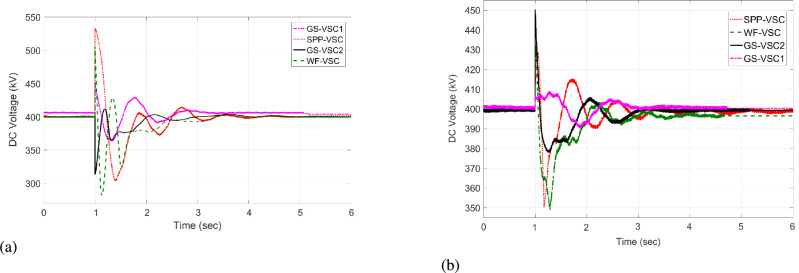
DC voltage response in case I; (**a**) VDC, and (**b**) HR-ADC.

**Fig. 18:**
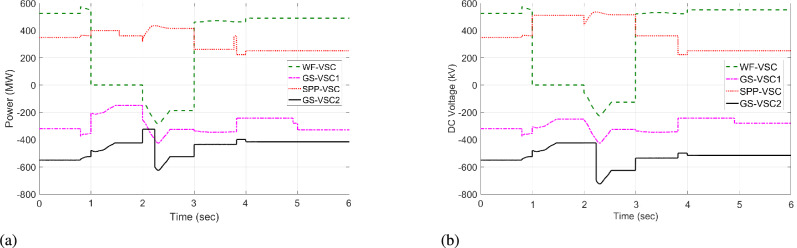
Response of power regulation in case I; (**a**) VDC, and (**b**) HR-ADC.

Case I is developed to evaluate the control effectiveness for regulating active power and DC voltage using VDC and HR-ADC in a significant power disturbance caused by WF VSC outage, which is illustrated in Fig 17 and Fig 18. During normal operational mode at t = 0 - 0.9 s, and t = 4 – 6 s, HR-ADC and VDC approaches work under uniform power and voltage states. As a result, the proposed objective is achieved in the MTDC grid. At t=1 s, WF VSC experiences a fault, resulting in the WF-VSC discontinuing supply power to the system and establishing unequal power distribution between rectifying and inverting VSCs. This fault introduces notable DC voltage fluctuation in MTDC. To address this issue, the SPP, $\hbox {GS}_1$, and $\hbox {GS}_2$ alter the control modes into the droop voltage and power modes from fixed DC voltage and power modes. Under this condition, VDC produces converter overloading problem with VSCs of SPP, $\hbox {GS}_1$, and $\hbox {GS}_2$ because of the power values of the droop coefficient values as provided in Eq. 8. This overloading causes overshoot of DC voltage of 530 and 479 kV for SPP-VSC and WF-VSC, as given in Fig 17a.

Whereas the proposed HR-ADC uses the designed droop coefficient values as present in Eq. 9 to modify the control mode into the droop power and voltage modes within the MTDC system. Under this condition, the SPP-VSC, $\hbox {GS-VSC}_1$, and $\hbox {GS-VSC}_2$ share and consume power based on the power values of the VSC, which improves unequal power allocation and DC voltage variation as given in Fig 17b and Fig 18b. After, some flexible deviations of DC voltage in VSC of WF and SPP, the grid keeps the regulation of balanced power that manages DC voltage at t = 3 s. The results confirm that an HR-ADC, by modifying droop coefficient values of WF-VSC, $\hbox {GS-VSC}_1$, and $\hbox {GS-VSC}_2$ and altering the control mode, effectively achieves proper power distribution and regulated DC voltage alternation. Thus, the ability to maintain power balance and stable DC voltage during the WF fault test condition validates the Hence, regulating the active power and DC voltage in the grid with the failure at WF validates the superior performance of the HR-ADC over VDC.

#### Case II: SPP converter terminal at fault

**Fig. 19:**
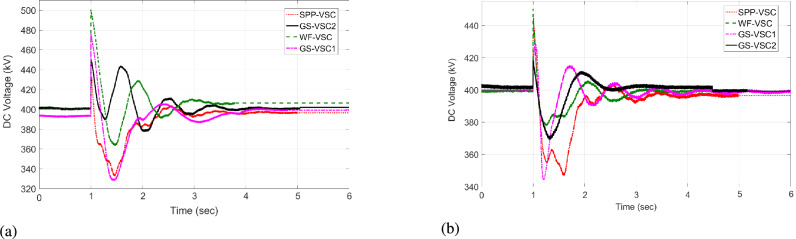
DC voltage response in case II; (**a**) VDC, and (**b**) HR-ADC.

**Fig. 20:**
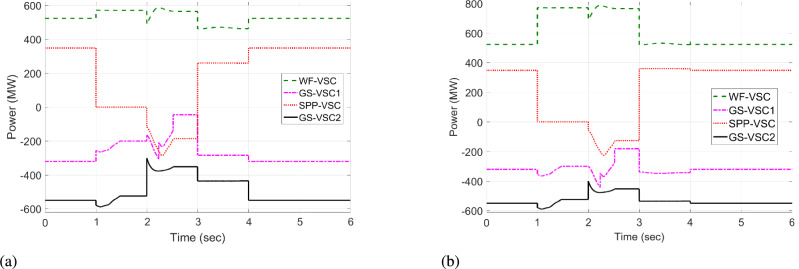
Response of power regulation in case II; (**a**) VDC, and (**b**) HR-ADC.

Similar to the case I, in case II HR-ADC and VDC function in the fixed voltage and power modes within steady state operational mode at t =0 - 0.9 s, and t = 4 – 6 s to stable DC voltage and regulate power allocation with MTDC as shown in Fig 19 and Fig 20. A fault at the converter terminal of SPP occurs at t=1 s and is cleared at t=3 s. During this condition, the VSC of SPP stops providing power to the MTDC system, causing unequal power distribution and generating DC voltage beyond stability limits.

During the fault at SPP converter terminal, VDC supplies 592 MW within the grid via WF-VSC and consumes power -600 and -300 MW via VSCs of $\hbox {GS}_1$ and $\hbox {GS}_2$ from MTDC system respectively at maximum power capacity of VSCs terminals based on the droop coefficient values as discussed in Eq. 8. This results in an overloading problem and leads to produce 500, 480, 460 and 460 KV DC voltage within system as illustrated in Fig 19a. The proposed HR-ADC utilizes droop coefficient values as outlined in Eq. 10 to switch operational states of fixed power and voltage to droop power and voltage modes. It leads to VSCs of WF, $\hbox {GS}_1$, and $\hbox {GS}_2$ sharing and consuming power based on the specified droop coefficient. This adaptive approach improves the unequal power distribution and DC voltage variations as given in Fig 19b and Fig 20b. Following slight fluctuations in DC voltage at the SPP and $\hbox {GS}_1$ terminals, the system restores balanced power allocation and stabilizes the DC voltage by t = 3 s. These results confirm that the HR-ADC by dynamically adjusting the droop coefficient values of WF-VSC, $\hbox {GS-VSC}_1$, and $\hbox {GS-VSC}_2$, accomplishes appreciated power distribution and regulation of DC voltage. Therefore, the outcomes of Case II validate the superior performance of HR-ADC compared to the VDC.

#### Case III : Fault on the converter station of $\hbox {GS}_2$

**Fig. 21:**
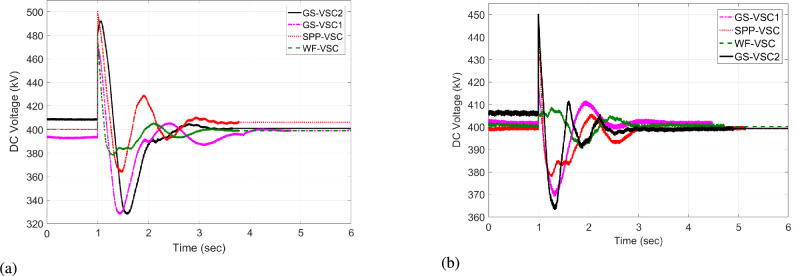
DC voltage response in case III; (**a**) VDC, and (**b**) HR-ADC.

**Fig. 22:**
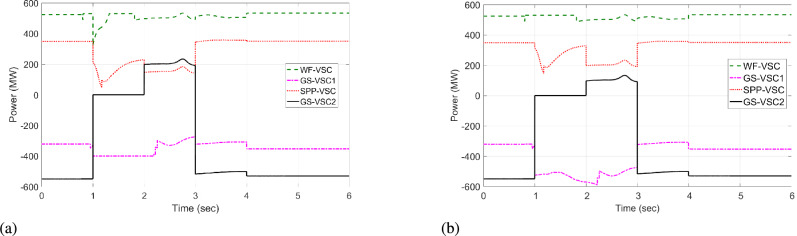
Response of power regulation in case III; (**a**) VDC, and (**b**) HR-ADC.

In case III, the proposed HR-ADC and VDC strategies perform well in the normal operational modes within MTDC grid from t = 0 -1 s, and t = 4 - 6 s, and accomplish the desired objective of maintaining DC voltage and keeping the equal power regulation with MTDC as given in Fig 21 and Fig 22, respectively.

This active power regulation is disrupted after at t = 1 s when the fault occurs at the VSC of $\hbox {GS}_{2}$. As a result, the $\hbox {GS-VSC}_{2}$ ceases importing power from the DC system and produces improper power regulation, resulting in substantial DC voltage change. The resulting power imbalance is redistributed among converter terminals of the VSCs of GS1, WF, and SPP. Under this disturbance, in VDC scheme causes overloading in $\hbox {GS}_{2}$, $\hbox {GS}_1$, and SPP due to the use of rated power values from t = 1 to 3 s, resulting in the 510, 485, and 500 kV DC voltage within network, respectively, as provided in Fig 21a and Fig 22a. However, the proposed HR-ADC during this scenario uses the designed droop coefficient, and $\hbox {GS-VSC}_2$ consumes -600 MW of power from the MTDC, which improves the power distribution and DC voltage fluctuation within the network, as shown in Fig 22b. Therefore, $\hbox {GS}_1$, $\hbox {GS}_{2}$, and SPP produce 450, 448, and 446 kV DC voltage as highlighted in Fig 21b. After some variations in the DC voltage levels of converter stations of $GS_{2}$, SPP, and $\hbox {GS}_1$ the grid keeps the regulation of balanced power that manages DC voltage at t = 3 s. The simulated results validated that the designed HR-ADC implemented in VSCs of WF, SPP, and $GS_{1}$ with adjusting operating modes using the intended droop coefficient values has resulted in sustained proper power balance, sustaining DC voltage within MTDC grid. Comparisons of results indicate that the HR-ADC provides effective performance in regulating power and DC voltage within the network under failure at $\hbox {GS}_2$-VSC.

## Conclusion

This study proposes an HR-ADC to eliminate the DC voltage deviation and sustain proper power distribution within MTDC. The available headroom of inverting and rectifying converters is utilized to design the droop coefficient for adjusting operational modes in the MTDC grid during the converter station outages or failures. To validate the proposed HR-ADC, a ± 400 kV four terminal MTDC grid was modeled in PSCAD, incorporating VSCs for WF, SPP, and grid side converters. Numerous scenarios, including the outages of VSCs at SPP, WF, $\hbox {GS}_1$, and $\hbox {GS}_2$ along with a fault on the WF, SPP, and $\hbox {GS}_2$ converters were simulated to evaluate the effectiveness of the system in meeting its control objectives of DC voltage control and maintain active power.

The simulation results of converter outage and fault demonstrated that the HR-ADC worked well within MTDC network. The DC voltage was kept under the safe operational range, and power regulation was successfully maintained across the network. Compared to VDC, the designed HR-ADC approach considerably improved balanced power utilization between rectifying and inverting VSCs and enhanced overall DC voltage stability. Additionally, root locus analysis was used to verified the stability of the proposed HR-ADC. The HR-ADC provides several valuable benefits. It improves DC voltage regulation by adjusting the droop coefficient according to the available headroom of rectifying and inverting VSCs, ensures balanced power allocation while avoiding overloading, and operates without any communication links. Collectively, HR-ADC improves the reliability, stability, and expandability of MTDC system relative to conventional droop based methods. However, suggested HR-ADC also has several limitations, as the control relies on accurate estimation of each VSC’s headroom and available power. In extreme multi-contingency situations, the controller may reach its saturation levels, limiting its capability to completely suppress voltage deviations. Moreover, its decentralized nature does not allow for a coordinated system, such as loss minimization or power flow optimization. Future developments can be related to addressing these challenges to further enhance control performance.

## Data Availability

The data supporting this study’s available to the corresponding author upon request.
